# Polarization coincidence images from metasurfaces with HOM-type interference

**DOI:** 10.1016/j.isci.2022.104155

**Published:** 2022-03-24

**Authors:** Tsz Kit Yung, Jiawei Xi, Hong Liang, Kai Ming Lau, Wai Chun Wong, Randy Stefan Tanuwijaya, Fan Zhong, Hui Liu, Wing Yim Tam, Jensen Li

**Affiliations:** 1 Department of Physics, The Hong Kong University of Science and Technology, Clear Water Bay, Hong Kong, China; 2 School of Physics, Southeast University, Nanjing 211189, China; 3 National Laboratory of Solid State Microstructures & School of Physics, Collaborative Innovation Center of Advanced Microstructures, Nanjing University, Nanjing 210093, China

**Keywords:** Physics, Photonics, Metamaterials

## Abstract

Metasurfaces provide a promising route for structuring light and generating holograms with designed amplitude, phase, and polarization profiles, leading to a versatile platform for integrating and constructing optical components beyond the conventional ones. At the same time, incorporating coincidence in imaging allows a high signal-to-noise ratio for imaging in very low light levels. As beneficial from the recent development in both metasurfaces and single-photon avalanche diode (SPAD) cameras, we combine the polarization-sensitive capability of metasurfaces with Hong-Ou-Mandel (HOM)-type interference in generating images with tailor-made two-photon interference and polarization coincidence signatures. By using orthogonal linear-polarized photons as incidence, correlated, anticorrelated, and uncorrelated polarization coincidence features can be observed within the same image from the pairwise second-order coherence statistics across different pixels of the image. Our work adds polarization to the demonstrated amplitude and phase sensitivity in the domain of “HOM microscopy” and can be useful for biological and security applications.

## Highlights

• Images with tailor-made two-photon interference and coincidence signatures are generated
• Control of spatial coincidence signal can be either on transmitted or diffracted fields
• The coincidence’s response can be correlated, anticorrelated, or uncorrelated
• The generation of coincidence images is potentially useful for quantum imaging schemes

## Graphical abstract

**Figure:**
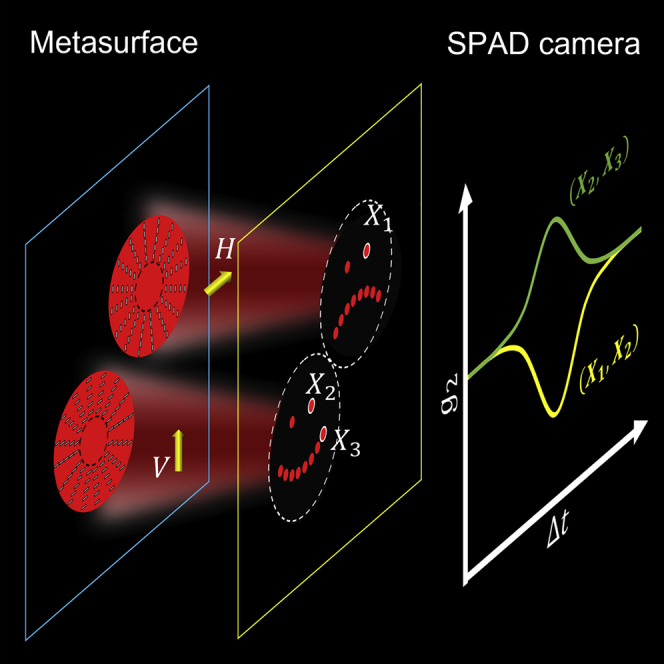

## Teaser

Physics; Photonics; Metamaterials; Hong-Ou-Mandel effect

## Introduction

Coincidence imaging was first introduced in a ghost-imaging setting in which a camera collects signals correlating with another bucket detector, allowing imaging with a high signal-to-noise ratio in very low light levels (Bennink et al. 2004; Erkmen and Shapiro, 2008). As the formation of a ghost image is purely due to the correlation between two propagation channels, the laser source used in the setup can be either classical (Bennink et al., 2002; Ferri et al., 2005) or quantum optical (Pittman et al., 1995; Chan et al., 2009), with the requirement that the input fields for the two arms of the imaging setup are in correlation. To prepare such correlation, a common method is to use quantum-entangled photon pairs from spontaneous parametric down-conversion (SPDC) in a nonlinear crystal. This correlation can be on positions, momenta, and arguably more complex structural illuminations (Padgett and Boyd, 2017). Interestingly, another important type of entangled state can be generated from HOM interference, with the entanglement involving the photon number. It has been recently shown that the resulting bunching of photon pairs can be used for quantum imaging by turning on or off the HOM effect using reflection/transmission amplitude or phase delay at different locations of the object to be imaged (Ibarra-Borja et al., 2020; Ndagano et al., 2021). Together with the recent advance in the increase of detection efficiency and the number of pixels of single-photon cameras in probing such coincidence, it is possible to measure coincidence between any two locations within the image when a SPAD camera is used. We are witnessing the developments of emerging imaging techniques in exploiting the temporal information of individual photons, pointing to applications including lidar-based 3D imaging, and a range of biophotonic or security applications (Zappa et al. 2007; Shin et al., 2016; Chrapkiewicz et al., 2016; Bruschini et al., 2019; Morimoto et al., 2020), which request the least number of photons or robustness against environmental noise.

Nanostructured metasurfaces allow manipulation of the different degrees of freedom of light, including amplitude, phase, polarization, and orbital angular momentum (OAM), with the finest resolution. They have been extensively used in miniaturizing, integrating, or obtaining functionalities beyond those of conventional optical elements (Hu et al., 2021). Examples include aberration-free metalenses (Wang et. al., 2018b; Chen et al., 2019), holograms (Zheng et al., 2015; Ni et al., 2013; Wen et al., 2015; Xiao et al. 2015, 2017; Zhao et al., 2018; Bao et al., 2021), polarization cameras (Arbabi et al., 2018; Rubin et al., 2019), and 3D light-field imaging (Lin et al., 2019; Guo et al., 2019). In the single-photon or low light regime, metasurfaces can already be designed as bandpass filters to reconstruct color images (Shah et al., 2020). The ability of metasurfaces in generating polarization-dependent images is currently leading to new opportunities in quantum imaging. For example, by entangling with a heralding photon, the two independent images can be superimposed through quantum entanglement and obtained respectively by projecting heralding photons onto different states, with applications on quantum edge detection and imaging with entangled photons (Zhou et al., 2020; Altuzarra et al., 2019). Nevertheless, coincidence in these cases is only measured between the signal (imaging) photons and the heralding photons in deciding whether the detected photons on the image should be recorded or not. For the case with interference, metasurface-based HOM interference has been demonstrated using non-classical light, including the use of multiphoton interference for state reconstruction (Wang et al., 2018a), and the demonstration of metasurface interferometry with polarization-entangled photons (Georgi et al., 2019) and spatial modes (Li et al., 2021). The possibility of tunable two-photon interference is also investigated in (Estakhri and Norris, 2021) by introducing reconfigurable metasurface using phase-change materials.

**Figure 1:**
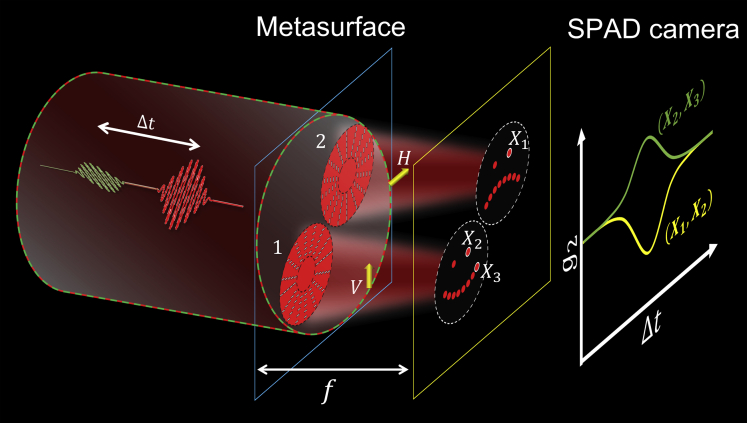
Coincidence images from a metasurface The metasurface, for example, consists of a pair of polarization lenses focusing light to two groups of focal points (holograms) with one in V polarization and another in H polarization at a distance of *f* away from the metasurface. It is then imaged by a SPAD camera. Two photons with orthogonal linear polarizations (colored in green and red) arrive at the metasurfaces with a tunable time delay Δt. The coincidence signal *g*_2_, obtained from the SPAD camera, between any pair of pixels on the image (e.g., between *X*_1_ and *X*_2_) can be plotted against Δt.

In this work, we combine the polarization-sensitive capability of metasurfaces with HOM-type interference in generating images with tailor-made two-photon interference and coincidence signatures. Such combination will be potentially useful for further developing quantum imaging schemes, e.g. Ndagano et al., (2021), in the future, or can be further extended to use tunable metasurfaces (Estakhri and Norris, 2021). Figure 1 shows our scheme of generating coincidence images from a metasurface, which is applicable with either a single-photon source (quantum) or phase-randomized weak coherent light source (classical) as we will develop and show toward the last part of the current work. Here, the term “coincidence images” means the image generated in having tailor-made coincidence signature. Two photons with diagonal $\left|D\right\rangle \left(+45{}^{\circ}\right)$ and antidiagonal polarization $\left|A\right\rangle \left(-45{}^{\circ}\right)$ at the same frequency arrive the metasurface with a tunable time delay Δt. The metasurface consists of two regions of nanostructures, labeled as the left disk |1⟩(right disk|2⟩) in generating an image in vertical polarization |V⟩(horizontal polarization|H⟩), and the remaining area does not transmit. The images are schematically shown as the two emojis on the plane at a distance *f* from the metasurface. The whole image is finally projected by a polarizer at 45° and taken by a time-resolved photon-counting SPAD camera.

Specifically, the two incident photons are prepared to hit the left and right disks with equal chance on the metasurface in transmission. In the case that the two photons arrive at the metasurface at the same time, the incident two-photon state (with boson symmetrization and normalization omitted for brevity) can then be written as

(Equation 1) $$\left(\left|1\right\rangle +\left|2\right\rangle \right)|D\rangle ⊗\left(\left|1\right\rangle +\left|2\right\rangle \right)|A\rangle$$

where $\left|D\right\rangle =\left(\left|V\right\rangle +\left|H\right\rangle \right)/\sqrt{2}$ and $\left|A\right\rangle =\left(\left|V\right\rangle -\left|H\right\rangle \right)/\sqrt{2}$. Because the left (right) disk is designed to pass only vertical (horizontal) polarization, applying projection operator |1V⟩⟨1V|+|2H⟩⟨2H| on each photon gives the output state as

(Equation 2) (|1V⟩+|2H⟩)⊗(|1V⟩−|2H⟩)

We have overloaded the symbols |1⟩ and |2⟩ to represent the field (amplitude and phase) profiles generated from the corresponding disks. The metasurface effectively entangles the polarizations with the image profiles |1⟩ and |2⟩ (the two emojis in Figure 1). In terms of operator representation, we write $\left|1V\right\rangle \to a_{1}^{†}\left|0\right\rangle$ and $\left|2H\right\rangle \to a_{2}^{†}\left|0\right\rangle$ using corresponding creation operators on the vacuum state |0⟩. Then, the output state can be rewritten as

(Equation 3) $$\begin{array}{l} \left(a_{1}^{†}+a_{2}^{†}\right)\left(a_{1}^{†}-a_{2}^{†}\right)\left|0\right\rangle =\left(a_{1}^{†}a_{1}^{†}-a_{2}^{†}a_{2}^{†}\right)\left|0\right\rangle \end{array}$$

In deriving Equation (3), we have assumed $a_{1}^{†}$ for the left ring approximately commutes with $a_{2}^{†}$ for the right ring, implying the missing of the $a_{1}^{†}a_{2}^{†}$ term, i.e., the HOM destructive interference. It means the second-order coherence *g*_2_ drops to zero. We note that in this work, we have actually used weak coherent states as the input states. The corresponding formulation starting from coherent states (see details in STAR methods) will pose a lower bound of *g*_2_ as 0.5 to achieve experimentally. We further note that here the images with HOM interference are obtained via the design of the metasurface. It can be via the capability of tailoring either spatial modes and also polarization in this work (Li et al., 2021; Estakhri and Norris, 2021). In terms of the second-order coherence *g*_2_ (termed as coincidence signal hereafter) between a pixel in the left image and another pixel in the right image, we expect to observe a dip in scanning Δt (e.g., anticorrelated photon between *X*_1_ and *X*_2_ in Figure 1). For $g_{2}$ between two different pixels within the same disk, a peak in *g*_2_ against Δt (e.g., correlated photon between *X*_2_ and *X*_3_) is then expected (from Equation 3). Therefore, for a fixed Δt and a chosen reference pixel, we can also plot the coincidence signal for each pixel with respect to the reference pixel as a “coincidence image”, in which correlated, anticorrelated, and uncorrelated polarization coincidence can be possibly observed all in a single image. To get a coincidence image with the largest contrast, we will choose Δt to have the strongest two-photon interference effect. Such an optimal Δt (conventionally defined as 0) has a similar role to the location of the image plane in getting the sharpest image in conventional imaging.

## Results

### Metasurface design and characterization

**Figure 2:**
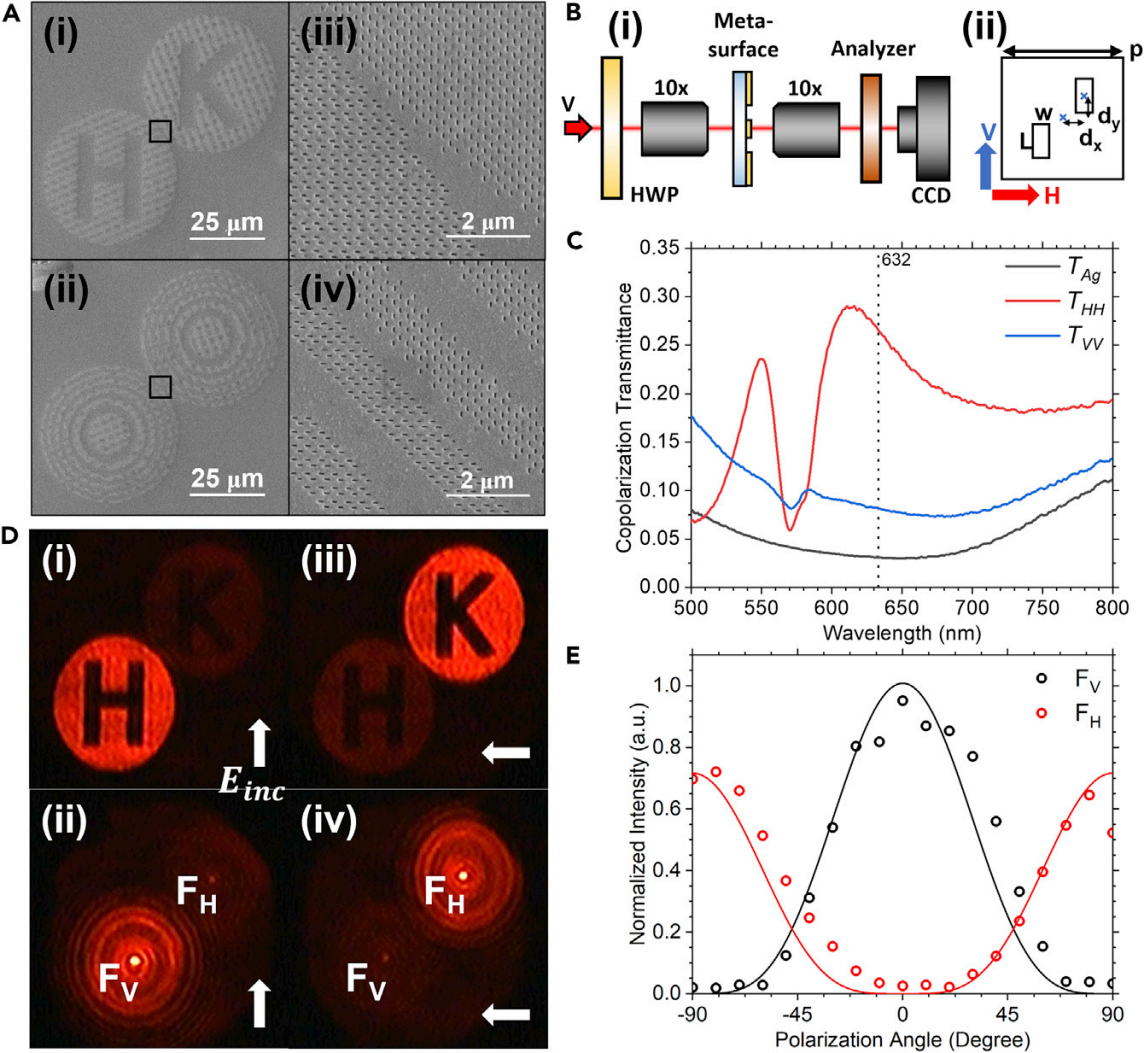
Characterization of metasurface in the classical regime (A) SEM images of the samples, consisting of metallic vertical and horizontal parallel nanoslots metasurfaces, (i) with “H”/“K” letters and (ii) with Fresnel zones for a 75 μm focal length. The regions encrypted in black boxes in (i) and (ii) of metasurfaces are enlarged in (iii)–(iv). (B)(i) Schematic of the experimental setup for sample characterization. A vertically polarized 632 nm laser beam is focused onto the metasurface to observe the change in image intensity versus incident polarization angle. The polarization axis of the analyzer is the same as incident polarization to enhance the intensity contrast for co-polarization measurements. (ii) The unitcell of basis structure used in (A). The horizontal and vertical polarization incident directions used in (C) are defined in red and blue arrows. (C) Measured co-polarization spectral response of the basis structure defined in (B)(ii). The red and blue curves are the transmission spectra for the horizontal and vertical polarization incidence, respectively. The black curve is for a 50 nm thick silver film. (D) CCD Images for the two metasurfaces in (A) when the incident polarization is vertical (0°), (i) and (ii), and horizontal (+/− 90°), (iii) and (iv), respectively. (E) Intensity versus incident polarization direction at the two focal points $F_{V}$ and $F_{H}$ as labeled in (D).

In this work, we image two polarization metasurfaces as shown in Figures 2A(i)–(ii) and zoom-in images (marked by the black rectangle boxes) in Figure 2A(iii)–(iv). The first sample consists of two circular disks (diameters 50 μm) of orthogonal (here horizontal and vertical) parallel nanoslots metasurface fabricated on a 50 nm thick Ag film by focused ion beam technique. Note that letters “H” and “K” are embedded, separately in the disks of the metasurface in Figure 2A(i), to demonstrate coincidence images using our setup. The second sample, in addition to having the same orthogonal metasurfaces (but without the “H”/“K” letters) as the first sample in Figure 2A(i), contains Fresnel zones in form of concentric rings as shown in Figure 2A(ii) with ring radii expressed as (Mote et al., 2008):

(Equation 4) $$r_{n}=\sqrt{n\lambda f+n^{2}\lambda^{2}/4}$$

for integer *n*, wavelength λ, and focal length *f*. The structure is fabricated on odd-numbered Fresnel zones (total 6) with 50 μm diameter for the outermost ring, giving a focusing length of 75 μm for wavelength 632 nm. The second sample combines the polarizing capability of metasurfaces with focusing effect from asymmetric constructive and destructive interference of the odd and even Fresnel zones with the first zone to enhance the sample’s transmission responses, producing a bright spot at the focal point. While the two focal spots are used here as an illustrative example, it is straightforward to use the same approach to generate holograms of more complicated structures.

Figure 2B(i) shows the setup for the characterization of the metasurfaces with conventional polarization optics as the first step. A vertically polarized 632 nm laser is used with a half-wave plate (HWP) to set the polarization of the incident light, while an analyzer with an optical axis aligned to the incident light enhances the contrast for sample imaging using a CCD camera when the polarization is varied. Figure 2B(ii) shows the unit cell of the basis structure used in Figure 2A. It consists of vertical parallel nanoslots with slot length L=125 nm, slot width w=50 nm, periodically placed in a square array with x&y period p = 330 nm, and slot x&y displacement *d*_*x*_ = *d*_*y*_ = 67.5 nm from the center of the unit cell. The horizontal and vertical polarization incidence direction used in (c) is defined in red and blue arrows. In comparison to Figure 2A(i), the unit cell of metasurface used in the “K” disk is shown in Figure 2B(ii) and the one used in the “H” disk is a 90° rotated version of Figure 2B(ii). Figure 2C shows the measured co-polarization transmittance for the basis structure shown in Figure 2B(ii). As the metallic nanoslots behave like a dipole at resonance in visible range (Park et al., 2018), it responses to incident polarization that is orthogonal to the slot direction (here horizontal, H). The corresponding transmission spectrum (red curve) has two resonance peaks as shown in Figure 2C. The first resonance peak at ∼540 nm is due to the excitation of first-order surface plasmon polariton (SPP) on the glass-Ag interface (Tavakoli et al., 2019). The coupling between the SPP and the slot cavity results in a higher transmission for the horizontal (H) polarization. The second resonance peak at ∼610 nm is due to the excitation of localized surface plasmon (LSP) from the slot cavity and hence is sensitive to the slot length (Lin et al., 2009). As a comparison, the transmission spectra for the vertical polarization incidence (blue curve) and a 50 nm thick silver film (black curve) are also included as reference in Figure 2C. At 632 nm (vertical dotted line), the ratio of transmittance between the two polarizations (red and blue curves) is around 3.5 times, confirming the polarizing capability of the slot structures, which are used to assemble the two metasurfaces in Figure 2A. The incident beam is focused to cover the fabricated patterns (the two disks) experimentally. For a 50 μm diameter “H”/”K″ disk pattern and a 100 μm diameter incident beam, the area ratio gives a factor of 0.25. Combined with the measured transmission efficiency of the structures in Figure 2C (0.27 at 632 nm) and the filling fraction of the structures in the samples, the final transmission efficiency is around 5.1% and 3.7% for the “H”/“K” sample and the Fresnel zone sample, respectively. Note that the transmission efficiency can be further improved by using dielectric metasurfaces.

Figures 2D(i)/(ii) and (iii)/(iv) show transmission images of the metasurfaces (Figure 2A) taken by the CCD camera for the vertical and horizontal polarization incidence, respectively. Overall, the results confirm both the polarization function of the metasurfaces and the focusing effect for the metasurface with Fresnel zones. Figure 2E shows the polarization dependence of the focal point (F_V_/F_H_) intensity, obtained from the gray-level image in Figure 2D versus incident polarization direction. The solid curves in Figure 2E are cos^4^ and sin^4^ fits of the data using the Jones matrix approach. The results show good polarization discrimination between the highest and lowest values for the samples, demonstrating the functionality of our metasurfaces. The small difference in the amplitudes and the offset in polarization angles in Figure 2E is due to imperfections in the fabrication.

### Photon-counted images of metasurface with SPAD camera

**Figure 3:**
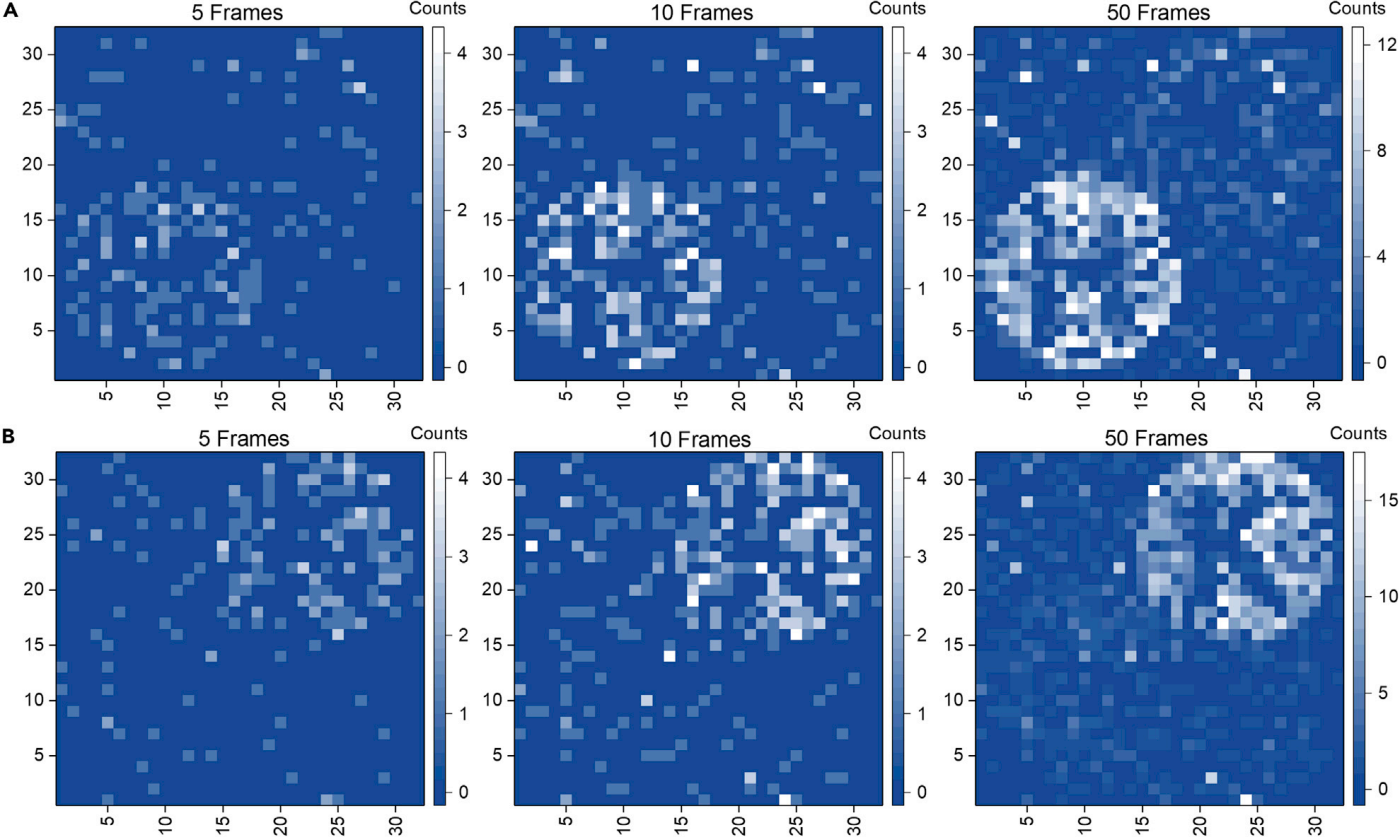
Photon counting image of a metasurface in the low light regime (A and B) The measurement is performed on the “H”/“K” lettered parallel nanoslots metasurface with (A) vertical polarization incidence and (B) horizontal polarization incidence. By summing the signal over multiple frames, the sample image starts to form when the detected photon number reaches a few thousand as shown in the middle column images.

To demonstrate the imaging capability of our system, we use an SPAD camera (Photon Force PF32) consisting of 32 × 32 pixel elements with 55 ps temporal resolution for our metasurface’s photon counting and coincidence measurement. Each pixel element, size 50 μm × 50 μm, has an active area of 6.95 μm in diameter and quantum efficiency of ∼20% at 632 nm. Figure 3 shows the single-photon count image of the “H”/“K”-lettered metasurface measured by the SPAD camera with frame length 2 μs using the setup in Figure 2B. The image of the metasurface, a lettered disk with a size ∼160 pixels, starts to appear after summing 5 frames’ signal and becomes clear for 10 frames, corresponding to an average of a few photons detected at each pixel. The details of the counting process are mentioned in the Method session. Summing all the photons arrived, the number of photons for the 5, 10, and 50 frames give 192, 413, and 1840 for the vertical polarization incidence and 203, 422, and 2193 for the horizontal polarization incidence. The exact dependence of total photon count versus accumulated frame number is included in the Supplemental information as reference. The small difference in photon counts for the same frame mainly comes from polarization axis mismatch due to fabrication error. As a comparison, a typical CCD’s image requires several order larger photon number to form, as estimated from the incident laser power and the exposure time of the CCD camera.

### Imaging metasurfaces using two-photon coincidence

Instead of using a quantum source (e.g., quantum dot or SPDC source) to generate two photons, we use a weak coherent light source with phase randomization in generating HOM-type interference (Kim et al. 2013, 2021; Zhang et al., 2020; Chen et al., 2016). When the intensity is low enough, such a classical light source mimics well the two-photon statistics. The adoption of a classical light source using phase-randomized weak coherent states provides relatively larger power available so that the exposure time can be shortened in the experiment, but with lower visibility of the quantum interference signal. Such an approach in using a classical light source in revealing quantum signature is also found useful in other applications like long-range quantum key distribution (Lo et al., 2012; Yin and Chen, 2019).

**Figure 4:**
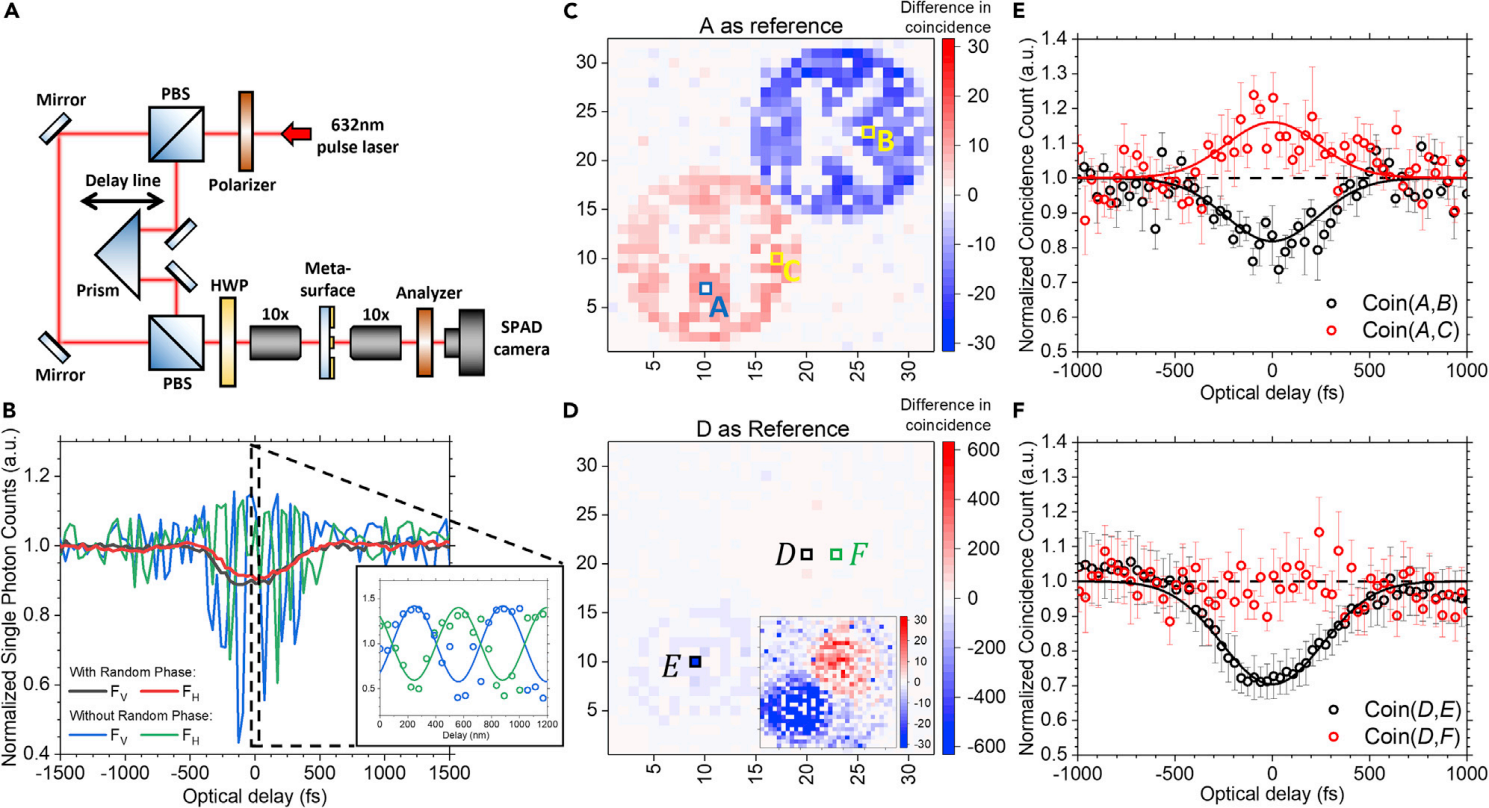
HOM-type interference with weak coherent pulses (A) Schematic of the experimental setup for the coincidence measurement. (B) Measured single-photon count for the metasurface with Fresnel zones at the focal point with and without phase randomization. The inset shows the classical interference oscillations in expanded scales (with value one renomalized as the mean count). (C and D) Coincidence images for the metasurfaces with “H”/“K” letters and Fresnel zones, respectively, are obtained by subtracting the image without quantum interference from that with quantum interference. Pixels D and E (in black open boxes) are focal points of the metasurface with Fresnel zones. A negative value in the coincidence difference implies a drop in the coincidence count. Pixel A (in blue open box) in (C) and pixel D in (D) is taken as the reference for computing the pairwise coincidence with all other pixels for the corresponding coincidence image. The inset in (D) plots the figure with the same scales as (C). (E and F) The second-order coherence statistics *g*_2_ for pixel pairs selected from the metasurfaces in (C) and (D). Data are represented as mean ± SEM.

Figure 4A shows the experimental setup for coincidence measurement. The incident laser beam is split into two with one beam passing through a motorized optical delay line and combined with another beam by a polarization beam splitter. The HWP in front of a 10x objective sets the incident polarizations of the beams, separately, to the diagonal (D) and anti-diagonal (A) directions. The laser spots for both polarizations are large enough to cover the whole area with nanostructures on the metasurface. After passing through the sample, the transmitted or diffracted beams will be focused by another 10x objective, projected by a polarizing analyzer at 45°, and finally focused on the SPAD camera for photon time-tagging. The phase randomization can be achieved by mechanical motion of optical components along one beam (Aragoneses et al., 2018). Specifically, the motor of delay line here in each step of motion unavoidably generates some residue vibration before the system is stabilized. It randomizes the phase of one beam in this period and the phase randomization is turned off after that. The characterization of delay line’s vibration is included in the Supplemental information as reference. For the metasurface with Fresnel zone, the single-photon count at the $F_{V}$ (blue curve) and $F_{H}$ (green curve) focal spots are plotted in Figure 4B without phase randomization. In this case, only classical interference is observed with oscillation against optical delay. The inset shows the details of this oscillation with expected period of laser wavelength 632 nm from a finer scan renormalized by the mean count. The deviation of the interference fringes from being symmetric about value one is due to the larger photon number at a pixel from the focused fields while the detectors only register the first arriving photon in the same frame. In contrary, when the phase randomization is on, the oscillation of the classical interference disappears, as displayed by the thick black (*F*_*v*_) and red (*F*_*H*_) curves in Figure 4B, leaving behind a shallow dip around zero Δt. In this case, two photons are bunched together to the same pixel, due to HOM quantum interference, which cannot be resolved by the SPAD camera at the same time. Such a drop of single photon count with phase randomization is connected to the asymmetric interference fringes without phase randomization (see details in STAR methods).

Figures 4C and 4D show the differential coincidence images for the two metasurfaces. Coincidence images are obtained by plotting the coincidence signal at each pixel with respect to a common reference (pixel A in Figure 4C and D in Figure 4D) at each Δt. Then, the differential coincidence image is obtained by subtracting the coincidence image without quantum interference (large Δt) from the one with quantum interference (zero Δt). A negative value (shown in blue) for a given pixel means anticorrelated photon (with respect to the reference pixel), while a positive value (shown in red) means correlated photon. Note that coincidence results between the reference pixel to itself are dropped to avoid having definite coincidence counts, hence pixels A and D appear white in Figures 4C and 4D. As shown clearly in Figure 4C, the differential coincidence image features well the letters as shown in Figure 2D, with correlated, anticorrelated, and uncorrelated polarization coincidence features observed within the same image. For the reference pixel A from the “H” image, as the two |1⟩ photons are bunched together (from Equation 3), a positive coincidence difference (correlated) is found for the “H” image. On the other hand, Equation (3) misses the $a_{1}^{†}a_{2}^{†}$ term due to HOM-type interference. For two-photon incidence, both of them will always exit the metasurface through the same ring (either left or right). It results in a much smaller chance to have the two photons coming through each ring separately (coincidence *g*_2_), compared to the case without quantum interference (*g*_2_ = 1). It is defined as a negative coincidence difference, i.e. photons arriving at one ring and at the other ring are anticorrelated. For Figure 4D, the coincidence difference at the focal point (pixel E) is much larger than those in Figure 4C due to focusing, as shown by the larger range in Figure 4D. When plotted in the same scale as Figure 4C, as displayed in the inset, similar polarization coincidence features are observed with swapped color due to the reference pixel being moved to the opposite disk.

Figures 4E and 4F are plots of the pairwise coincidence count versus the time delay  Δt for the metasurfaces with “H”/“K” letters and Fresnel zones normalized by that without interference (large Δt), respectively. The red and black symbols are the coincidence count for pixel pairs labeled in Figures 4C and 4D while the solid curves are Gaussian fits of the corresponding data to show the visibility of the data. For Figure 4E, the measured quantum constructive interference (co-polarization, *g*_2_ peak) in general, has visibility lower than that of the destructive interference (cross-polarization, *g*_2_ dip) due to a drop in the single-photon count and hence the coincidence count during interference, as mentioned before for Figure 4B. For Figure 4F, as the diameter of the focusing spot is comparable to the active area of the SPAD camera’s pixel, the interference effect vanishes quickly when one of the selected pixels in the pixel pair is away from the focal point, e.g., Coin(*D*, *F*), due to the absence of signal. The observed visibility for HOM-type interference is slightly increased with focusing due to the enhancement in coincidence’s signal-to-noise ratio when comparing Coin(*D*, *E*) with Coin(*A*, *B*). Note that as the source is a weak coherent state with phase randomization, the lower bound for the *g*_2_ dip is 0.5 (Kim et al. 2013, 2021). Here, the drop in HOM-type interference visibility is due to the deviation of the plasmonic slots from ideal polarizers, background transmission from silver film, and also small asymmetry in amplitude in preparing the two beams shining on the metasurfaces.

## Discussion

In conclusion, we combine the polarization-sensitive capability of metasurfaces with HOM-type interference in generating images with tailor-made two-photon interference and coincidence signatures. The metasurfaces provide control on the polarization-coincidence signal between different designated positions either on the metasurfaces or on the focal plane of the diffracted fields from the metasurface. The polarization coincidence signal response can be tuned to be either correlated, anticorrelated, or uncorrelated. Further generalization to other degrees of freedom (DOF) like OAM that support HOM-type interference is also possible. Our investigations also add polarization sensitivity to the coincidence measurement using a SPAD camera. We believe our work points to the usage of metasurfaces toward the preparation of entangled-photon-assisted imaging and generating entangled holograms as well.

### Limitations of the study

The current metasurface design has large transmission loss due to the use of plasmonic resonance; its performance can be improved by using a dielectric structure. Also, the use of coherence state introduces a bound in obtained coincidence count visibility which reduces the contrast of coincidence image; this can be improved by using a single-photon source instead.

## STAR★Methods

### Key resources table

**Table:** 

REAGENT or RESOURCE	SOURCE	IDENTIFIER
**Software and Algorithms**		

MATLAB	The MathWorks, Inc	R2020b

**Other**		

E-beam Evaporator	AST	600 EI Evaporator
Dual Beam FIB/SEM	FEI	Helios G4 UX
SPADs camera	Photon Force	PF32

### Resource availability

#### Lead contact

Further information and requests for resources and reagents should be directed to and will be fulfilled by the lead contact, Jensen Li (jensenli@ust.hk).

#### Materials availability

This study did not generate new unique reagents.

### Experimental model and subject details

The study does not use experimental models typical in the life sciences.

### Method details

The pulse laser used in the experiment is a 20 MHz 6 ps Fianium WhiteLase^TM^ micro supercontinuum mode-locked fiber laser. We select, through a monochromator, the 632 nm +/− 1.5 nm line as our light source. The pulse width is downgraded to 150 ps due to dispersions from the monochromator and the optical setup. When a photon strikes a pixel of the SPAD camera, the arrival time of the photon at this pixel will be time-tagged and this pixel will become inactive until the whole image is exported for storage at the end of each frame. Thus, each pixel can detect the first arrival photon for each frame, subsequent photons hitting the same pixel will be ignored. For the photon-counting image, the time-tag of photons is neglected and only the photon number is counted within the exposure time of the camera. For the coincidence image, the coincidence count for each pixel is first determined for each frame using a reference pixel, when the recorded photon pair’s arrival time is within a coincidence window (+-550 ps). For Figures 4C–4F, 100 k frames are taken at each time delay Δt for 500 ms exposure time. Summing all the coincidence signal (counts) at each Δt form a raw coincidence image, *RC*(*x*, *y*, Δ*t*) The image shown in Figures 4C–4D is obtained by averaging the raw coincidence image and then subtracting the averaged image without interference from that with interference, mathematically written as $1/5\sum_{i}^{5}RC\left(x,y,\;\Delta t_{i}∼0fs\right)-1/10\sum_{j}^{10}RC\left(x,y,\;\Delta t_{j}∼\pm 1000fs\right)$. For Figures 4E–4F, the plot is *RC*(*x_i_*, *y_i_*, Δ*t*) versus Δt for a given pixel (*x*_*i*_, *y*_*i*_) relative to the reference pixel, normalized by $1/20\sum_{j}^{20}RC\left(x_{i},y_{i},\;\Delta t_{j}∼\pm 1000fs\right)$ as the *g*_2_ should be 1 for large Δt. Note that the operation frequency of the SPAD camera is varied slightly with respect to that of the pulse laser to increase the signal-to-noise ratio of measured coincidence count.

### Quantification and statistical analysis

The study does not include statistical analysis or quantification.

### Additional resources

The study has not generated or contributed to a new website/forum or if it is not part of a clinical trial.

## Data Availability

• Data reported in this paper will be shared by the lead contact upon request. • This paper does not report original code. • Any additional information required to reanalyze the data reported in this paper is available from the lead contact upon request.

## Supplemental information

Document S1. Method details and Figures S1–S5
