# Supplementary material for: Polarization coincidence images from metasurfaces with HOM-type interference
Source: iScience. 2022 Mar 24;25(4):104155. doi: 10.1016/j.isci.2022.104155 (PMC9010753; doi:10.1016/j.isci.2022.104155)
Supplement: Document S1. Method details and Figures S1–S5 [file mmc1.pdf]

iScience, Volume *25*

Supplemental information

Polarization coincidence images from metasurfaces with HOM-type interference

Tsz Kit Yung, Jiawei Xi, Hong Liang, Kai Ming Lau, Wai Chun Wong, Randy Stefan Tanuwijaya, Fan Zhong, Hui Liu, Wing Yim Tam, and Jensen Li

Here gives the details on how HOM-type interference can be discussed from a product state of diagonal and anti-diagonal polarized coherent pulses using a coherent state formulation, in addition to the simpler picture from a two-photon state in main text. We let the two coherent input states as $|\alpha_D\rangle = \widehat{\mathcal{D}}_D(\alpha_D)|0\rangle$ for D polarization and $|\alpha_A\rangle = \widehat{\mathcal{D}}_A(\alpha_A)|0\rangle$ for A polarization with corresponding displacement operator $\widehat{\mathcal{D}}(\alpha) = e^{\alpha\hat{a}^\dagger - \alpha^*\hat{a}}$ defined from creation operator $\hat{a}^\dagger$. In the experiment, the metasurface is imaged by a SPADs camera. Consider the case where the metasurface is spatially divided into n output ports that pass a particular polarization direction, with each port having one pixel detector from the camera. In terms of the displacement operators, the operation of metasurface, together with the analyzing polarizer in front of the camera, can be written as

$$\widehat{\mathcal{D}}_D(\alpha_D)\widehat{\mathcal{D}}_A(\alpha_A)|0\rangle \to \prod_n \widehat{\mathcal{D}}_n(\alpha_n)\,|0\rangle, \tag{S1}$$

with output coherent state's amplitude α_n at the nth spatial port by assuming the creation operators for different output ports commuting with each other. Here, $\alpha_n = \alpha_{nD} + \alpha_{nA}$ with α_{nD}, α_{nA} being the complex amplitudes of coherent states arriving n^{th} port (pixel) from the incident D and A beams respectively. For brevity, we just write $|\alpha_D\alpha_A\rangle \to |\alpha_1\alpha_2 \ldots\rangle$ for Eq. S1.

When the light arrives two different pixels (i, j) through two disks of the metasurface, the output state is $\widehat{\mathcal{D}}_i(\alpha_i)\widehat{\mathcal{D}}_j(\alpha_j)|0\rangle$ from Eq.S1 with $\alpha_i = \alpha_{iD} + \alpha_{iA} = t_{iD}\alpha_D + t_{iA}\alpha_A$ and $\alpha_j = \alpha_{jD} + \alpha_{jA} = t_{jD}\alpha_D + t_{jA}\alpha_A$. ($\alpha_{iD} \triangleq t_{iD}\alpha_D$, $\alpha_{iA} \triangleq t_{iA}\alpha_A$, etc.) The t coefficients transfer the inputs (D & A polarizations) to the outputs. First, to have a qualitative understanding, we expand the displacement operators in Eq. S1 up to the lowest two orders (weak source) with $\hat{a}_D^\dagger \to \Sigma_i t_{nD}\hat{a}_n^\dagger$, $\hat{a}_A^\dagger \to \Sigma_n t_{nA}\hat{a}_n^\dagger$:

$$\begin{aligned}|\alpha_D\alpha_A\rangle &\cong \left(I + \alpha_D\hat{a}_D^\dagger - \alpha_D^*\hat{a}_D + \cdots\right)\left(I + \alpha_A\hat{a}_A^\dagger - \alpha_A^*\hat{a}_A + \cdots\right)|0\rangle \\ &\to (1 - \Sigma_n\alpha_{nD}^*\alpha_{nA})|0\rangle + \Sigma_n(\alpha_{nD} + \alpha_{nA})\hat{a}_n^\dagger|0\rangle + \Sigma_n\alpha_{nD}\alpha_{nA}\hat{a}_n^\dagger\hat{a}_n^\dagger|0\rangle \\ &\quad + \Sigma_i\Sigma_{j>i}\left(\alpha_{iD}\alpha_{jA} + \alpha_{jD}\alpha_{iA}\right)\hat{a}_i^\dagger\hat{a}_j^\dagger|0\rangle + \cdots.\end{aligned} \tag{S2}$$

The last term $\alpha_{iD}\alpha_{jA} + \alpha_{jD}\alpha_{iA}$ for the pixel i and j will vanish at Hong-Ou-Mandel (HOM) interference, leading to a drop in coincidence count between the two pixels. Now, we consider the photon statistics directly from the output coherent states α_i and α_j. Assume all the camera's

detectors have the same detection efficiency η and it does not distinguish photon number (i.e, only differentiate no photon and has photon in the same frame). The total probability for having photon detected at the pixel i and j are P_i and P_j:

$$P_i = \sum_{N=1}^{\infty} |\langle N_i|\alpha_i\rangle|^2(1-(1-\eta)^N) = 1-e^{-\eta|\alpha_i|^2}, \tag{S3a}$$

$$P_j = \sum_{M=1}^{\infty} \left|\langle M_j|\alpha_j\rangle\right|^2(1-(1-\eta)^M) = 1-e^{-\eta|\alpha_j|^2}, \tag{S3b}$$

with coincidence probability P_{ij} between different pixels i and j given by:

$$P_{ij} = \sum_{N=1}^{\infty}\sum_{M=1}^{\infty} \left|\langle N_i M_j|\alpha_i\alpha_j\rangle\right|^2(1-(1-\eta)^N)\,(1-(1-\eta)^M)\,. \tag{S4}$$

$|N_i\rangle$ and $|M_j\rangle$ are the Fock state denoting N and M photons at the pixel i and j. From Eq. S3, the single-photon count for pixel i normalized to ballistic one (superscript "b") is therefore derived as

$$\frac{P_i}{P_i^{(b)}} = \frac{1-e^{-\eta|\alpha_{iD}+\alpha_{iA}|^2}}{1-e^{-\eta(|\alpha_{iD}|^2+|\alpha_{iA}|^2)}}. \tag{S5}$$

which translates to the classical interference in the square amplitude $\frac{|\alpha_{iD}+\alpha_{iA}|^2}{|\alpha_{iD}|^2+|\alpha_{iA}|^2} \in [1-v_i, 1+v_i]$ with interferometric visibility $v_i = \frac{2|\alpha_{iD}||\alpha_{iA}|}{|\alpha_{iD}|^2+|\alpha_{iA}|^2}$ (usual MZ interferometer setting) at very small η. When there is a higher photon number to be detected by a pixel (in our case it can b from a highly focused field), the first order series expansion for the exponentials in Eq. S5 may not be enough, visualized as the interference fringes in Fig. 4b (0.43 to 1.16) being asymmetric about value 1.

From Eq. S4 and S5, the visibility on single-photon count $V_i \triangleq 1-\langle P_i\rangle/P_i^{(b)}$ and visibility on coincidence count $V_{ij} \triangleq 1-\langle P_{ij}\rangle/P_{ij}^{(b)} = 1-\langle P_{ij}\rangle/\left(P_i^{(b)}P_j^{(b)}\right)$ with phase randomization (denoted by angle bracket $\langle\ \rangle$) can be derived by ensemble averaging a uniformly random phase difference between the two incident beams α_D and α_A:

$$V_i = \frac{1-P_i^{(b)}}{P_i^{(b)}}(I_0(2\eta|\alpha_{iD}||\alpha_{iA}|)-1), \tag{S6}$$

$$V_{ij} = \frac{V_i}{P_j^{(b)}} + \frac{V_j}{P_i^{(b)}} - \frac{1-P_i^{(b)}}{P_i^{(b)}}\frac{1-P_j^{(b)}}{P_j^{(b)}}\Delta_{ij} \cong \frac{1}{2}\left(-\cos\arg\frac{\alpha_{jD}\alpha_{iA}}{\alpha_{iD}\alpha_{jA}}\right)v_i v_j \leq 0.5. \qquad \text{(S7)}$$

with I_0 being the zeroth order modified Bessel function of the first kind, $\Delta_{ij} \triangleq I_0\left(2\eta\sqrt{\left|\alpha_{iD}\alpha_{jA}+\alpha_{jD}\alpha_{iA}\right|^2+\left(|\alpha_{iD}|^2-\left|\alpha_{jD}\right|^2\right)\left(|\alpha_{iA}|^2-\left|\alpha_{jA}\right|^2\right)}\right)-1$, which evaluates to approximately zero when $|\alpha_D| = |\alpha_A|$ and HOM condition $t_{iD}t_{jA} + t_{jD}t_{iA} = 0$. The second equality in Eq. S7 is in the limit of small η. In Eq. S6, V_i will approach zero (flat single-photon count) only when the term $\eta|\alpha_{iD}||\alpha_{iA}| \to 0$, which corresponds to the case with a small detected photon number ($P_i^{(b)} \ll 1$). For the "HK" sample, this number is in the order of 0.1 to 0.2. For the Fresnel zone plate sample, the focused fields at the two pixels can be as high as 0.8. We have to use Eq. (S6) to evaluate the finite visibility. Physically, the single-photon count's visibility comes from the assumption that the detector cannot resolve the photon's number. When we have two or more photons falling onto the same frame onto the same pixel, the pixel can only record the first arrival photon and treat the number of photons of that particular pixel as 1 in this frame. This underestimates the photon count, in particular at the situation at HOM interference in which photons bunch together into the same output port and hence the same pixel has a much higher chance, i.e. giving a dip in the single-photon count. In Eq. S7, v_i is the (classical) interferometric visibility for the two polarizations arriving port i. $\arg\frac{\alpha_{jD}\alpha_{iA}}{\alpha_{iD}\alpha_{jA}}$ for our metasurface is designed to be π and the bracket term goes to value 1 at designed HOM condition of the metasurfaces. This imposes an upper bound of 0.5 on V_{ij} with ideal classical interferometric visibility $\left(v_i = v_j = 1\right)$ due to the contamination in coincidence from multiple-photons input (coherent state).

Experimentally, from the asymmetric interference fringes in Fig. 4b (0.43 to 1.16), v_i is experimentally found to be 0.74 from the bounds of Eq. S5. Using this value, assume the two pixels have similar statistics, the single-photon visibility V_i is then estimated to be 0.1 from Eq. S6 and coincidence visibility V_{ij} is estimated to be 0.28 from Eq. S7, which agrees with experimental results. Here, the drop in classical interferometric visibility (v_i, v_j) is mainly due to that the plasmonic slots as polarizers give an extinction ratio of around only 1:0.3 (Fig. 2c). To include this effect in the model (e.g. in using Eq. S7), suppose port i (j) passes V (H) polarization with t and

passes H (V) polarization with residue t'. The action of metasurface with analyzing polarizer at 45 degrees becomes:

$$\begin{pmatrix} \alpha_i \\ \alpha_j \end{pmatrix} = \frac{1}{2}\begin{pmatrix} t + t' & t - t' \\ t + t' & t' - t \end{pmatrix}\begin{pmatrix} \alpha_D \\ \alpha_A \end{pmatrix}. \tag{S8}$$

From the sample's extinction ratio, $|t'|^2/|t|^2 \cong 0.3$. By taking the relative phase between t' and t into account, the classical interferometric visibility is expected to lie between 0.53 and 1, agreeing to the estimated value from Fig. 4b. Other factors like the background transmission from Ag film and asymmetric local amplitude in preparing the D and A beams can also contribute and further decrease the visibility.

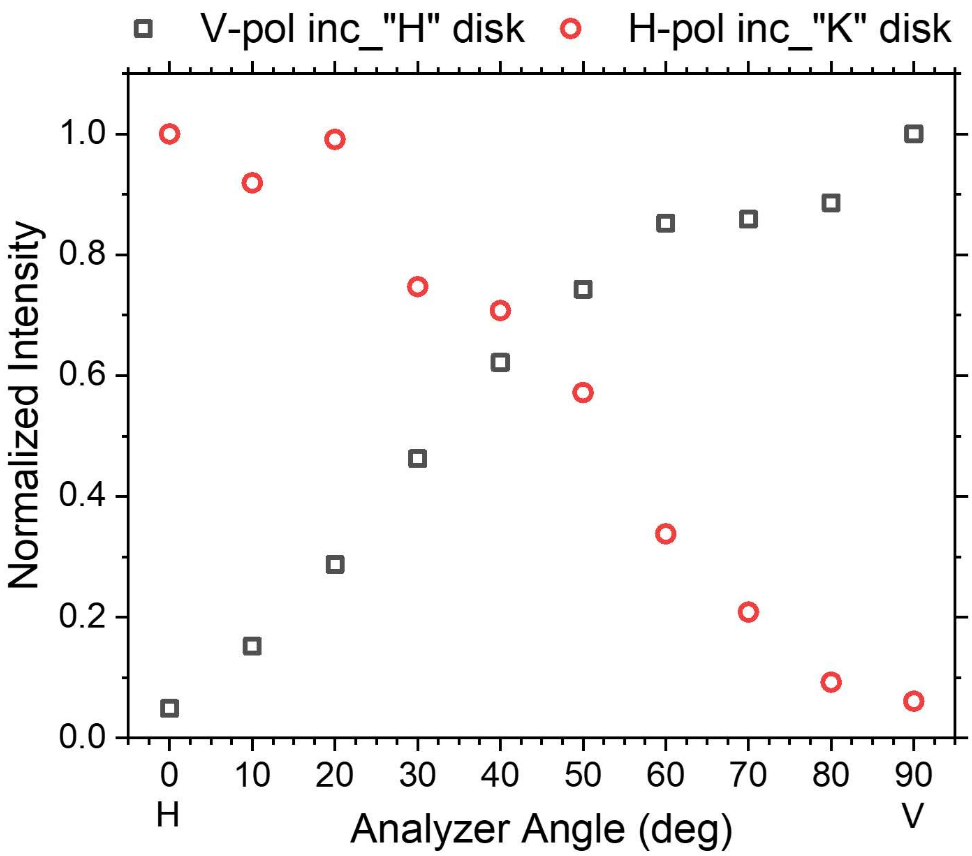

Figure S1. Cross-polarization measurement of "H"/"K" metasurface, related to Figure 2(c).

The scatters are the measured intensity for vertical polarization incident on "H" disk (Black) and horizontal polarization incident on "K" disk (red) as a function of analyzer angle (0deg: horizontal polarization, 90deg: vertical polarization). From the data, the cross-polarization intensity is ~5% of the co-polarization one, which is neglectable and hence is not included in Fig.2(c).

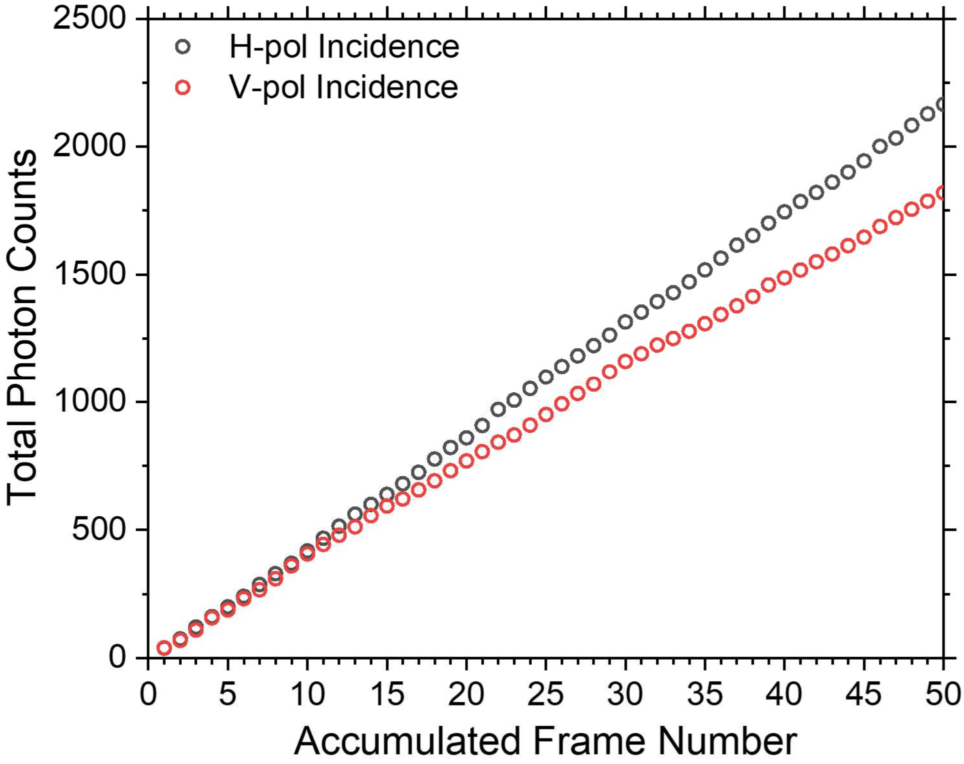

Figure S2. Photon accumulation curves obtained from Figure 3.

The scatters are calculated by summing all the pixel count from the camera for horizontal and vertical polarization incidence. The plots are approximately linear. The small slope difference between the two curves comes from the analyzer's polarization axis mismatch and the resultant transmittance difference in the experiment.

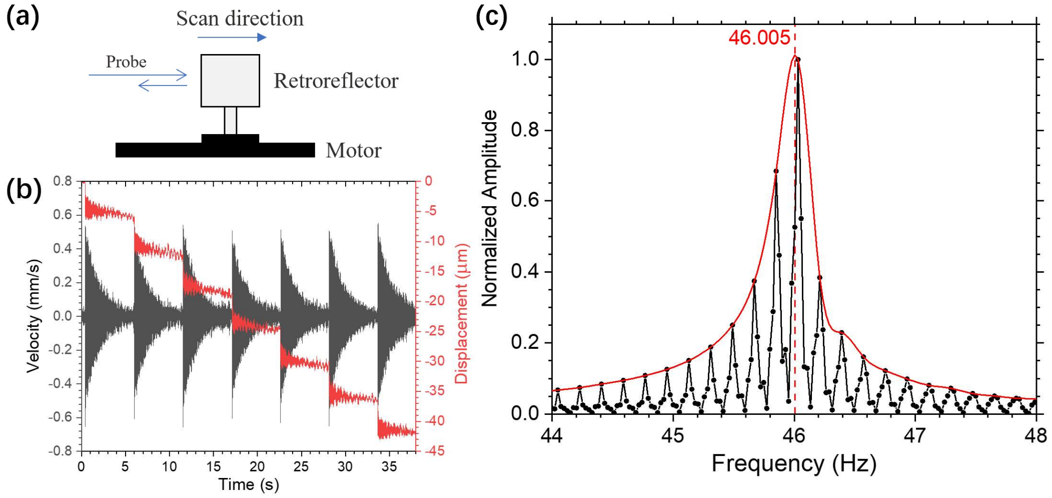

Figure S3. **Characterization of the phase randomization process used in Figure 4(a).**

a) Experimental Setup. The delay line's residual vibration is characterized by a laser doppler vibrometer (OptoMet SWIR SLDV) when the motor is assigned to move in 5μm step and wait for 5.5s for each step in the forward direction. b) Measured velocity (Black curve) and the measured displacement (Red curve) for a 7 steps scan. c) Frequency spectrum of the velocity oscillation in (b). The red curve is the upper envelope of the data. From the envelope, the delay line oscillates at ~46Hz with a bandwidth of 0.42 Hz. The black peaks are due to the artificial repeating period of the signal in (b).

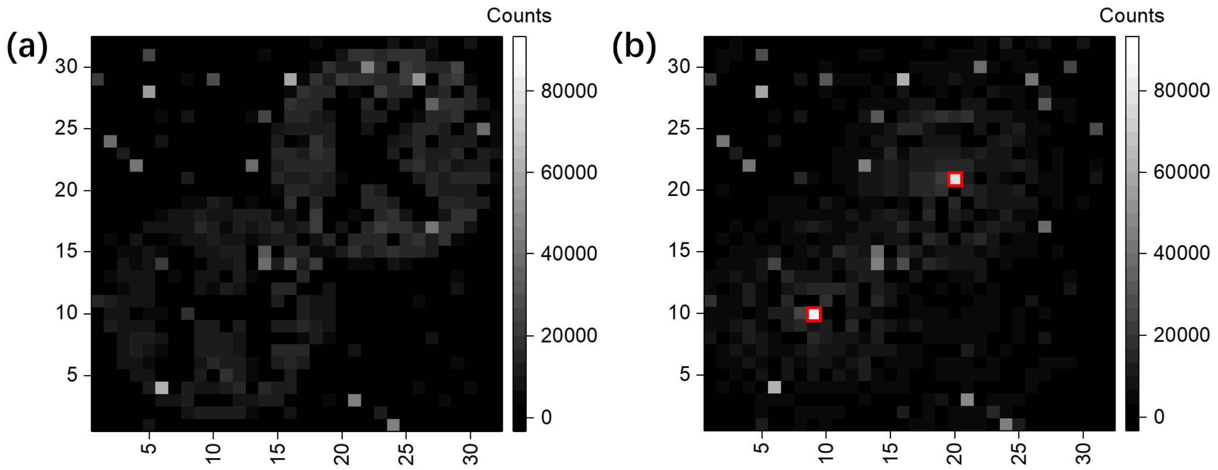

Figure S4. **Single-photon image as a comparison with Figure 4(c),4(d)**

Measured single-photon image (for 100k frames) using the setup in Figure 4 for a) “H”/”K” sample and b) Fresnel zone sample plotted in the same scale. The red squares in (b) indicate the sample’s focal points. By comparing (a) and (b), one can see that signal level at the two focal points of the Fresnel zone sample is much higher compared to the “H”/”K” sample due to focusing, leading to a significantly higher coincidence count in Fig. 4(d) relative to Fig. 4(c).

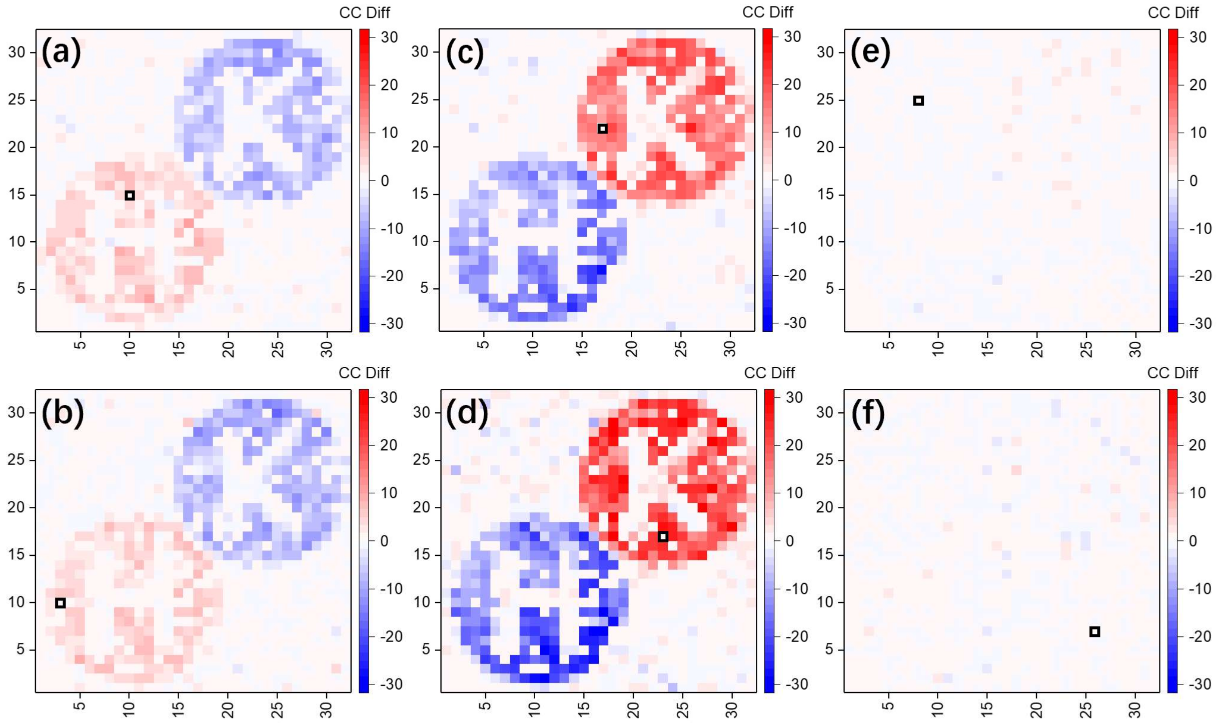

Figure S5. **Effect of change in reference pixel as a comparison to Figure 4(c).**

The coincidence image of “H” and “K” plot in coincidence difference (CC diff) with difference reference pixel (Black open square). Three types of images can be observed depending on the location of the reference pixel. (a)-(b) The reference pixel is located within “H”, giving a positive coincidence difference on “H” (red) and negative coincidence difference on “K” (blue) for co- and cross-polarization coincidence measurement respectively. (c)-(d) The reference pixel is located within “K”. The color of the image is flipped compared to (a)-(b) as co-polarization coincidence is found within “K” instead of “H”. (e)-(f) The reference pixel is located outside “H” and “K”. No feature is observed in this case.
